# Statins Reduces the Risk of Dementia in Patients with Late-Onset Depression: A Retrospective Cohort Study

**DOI:** 10.1371/journal.pone.0137914

**Published:** 2015-09-18

**Authors:** Ya-Hsu Yang, Hao-Wei Teng, Yen-Ting Lai, Szu-Yuan Li, Chih-Ching Lin, Albert C. Yang, Hsiang-Lin Chan, Yi-Hsuan Hsieh, Chiao-Fan Lin, Fu-Ying Hsu, Chih-Kuang Liu, Wen-Sheng Liu

**Affiliations:** 1 Department of Psychiatry, Taipei City Hospital, Renai Branch, Taipei, Taiwan; 2 National Yang-Ming University School of Medicine, Taipei, Taiwan; 3 Division of Hematology and Oncology, Department of Medicine, Taipei Veterans General Hospital, Taipei, Taiwan; 4 Department of Physical Medicine and Rehabilitation, National Taiwan University Hospital Hsin-Chu Branch, Hsinchu, Taiwan; 5 Department of Nursing, Yuanpei University, Hsinchu, Taiwan; 6 Division of Nephrology, Department of Medicine, Taipei Veterans General Hospital, Taipei, Taiwan; 7 Department of Psychiatry, Taipei Veterans General Hospital, Taipei, Taiwan; 8 Department of Child Psychiatry, Chang Gung Memorial Hospital and University, Taoyuan, Taiwan; 9 Division of Nephrology, Department of Medicine, Taipei City Hospital, Zhong-Xing Branch, Taipei, Taiwan; 10 College of medicine & Graduate institute of Business Administration, Fu Jen Catholic University, New Taipei city, Taiwan; National Health Research Institutes, TAIWAN

## Abstract

**Objective:**

Patients with late-onset depression (LOD) have been reported to run a higher risk of subsequent dementia. The present study was conducted to assess whether statins can reduce the risk of dementia in these patients.

**Methods:**

We used the data from National Health Insurance of Taiwan during 1996–2009. Standardized Incidence Ratios (SIRs) were calculated for LOD and subsequent dementia. The criteria for LOD diagnoses included age ≥65 years, diagnosis of depression after 65 years of age, at least three service claims, and treatment with antidepressants. The time-dependent Cox proportional hazards model was applied for multivariate analyses. Propensity scores with the one-to-one nearest-neighbor matching model were used to select matching patients for validation studies. Kaplan-Meier curve estimate was used to measure the group of patients with dementia living after diagnosis of LOD.

**Results:**

Totally 45,973 patients aged ≥65 years were enrolled. The prevalence of LOD was 12.9% (5,952/45,973). Patients with LOD showed to have a higher incidence of subsequent dementia compared with those without LOD (Odds Ratio: 2.785; 95% CI 2.619–2.958). Among patients with LOD, lipid lowering agent (LLA) users (for at least 3 months) had lower incidence of subsequent dementia than non-users (Hazard Ratio = 0.781, 95% CI 0.685–0.891). Nevertheless, only statins users showed to have reduced risk of dementia (Hazard Ratio = 0.674, 95% CI 0.547–0.832) while other LLAs did not, which was further validated by Kaplan-Meier estimates after we used the propensity scores with the one-to-one nearest-neighbor matching model to control the confounding factors.

**Conclusions:**

Statins may reduce the risk of subsequent dementia in patients with LOD.

## Introduction

Older people with late-life depression (LLD) are reported to run a higher risk of cognitive impairment [1] and dementia [2]. LOD is a subtype of depression occurring for the first time in later life (cut-off age range from 50 to 65 y/o)[3–5]. A close relationship between LOD and subsequent dementia has been reported [6–8]. In the EURODEM study, four of six case-control studies revealed that a positive relationship between depression and subsequent onset of Alzheimer's disease was confined to people with LOD (Odds Ratio [OR], 2.44; 95% confidence Interval [CI], 1.36–4.36)[9, 10]. This finding was further confirmed by a large-scale cohort study and meta-analysis[11]. Taken together, LOD is a risk factor or prodromal of further dementia; however, the medication in preventing the subsequent dementia in patients with LOD remains unclear.

Statins are a class of drugs that inhibit 3-hydroxy-3-methylglutaryl coenzyme A (HMG-CoA) reductase that catalyzes the rate limiting step in cholesterol biosynthesis to reduce levels of circulating cholesterol and also inhibit de novo cholesterol synthesis in the brain. Statins may reduce the risk of cerebrovascular disease by reducing atherosclerosis and improving cerebral perfusion. High dose atorvastatin has shown to reduce the risk of recurrent ischemic stroke among patients with an initial stroke or TIA after a mean follow-up of 4.9 years [12]. Statins are considered as the first-line agents for treatment of hypercholesterolemia for the primary and secondary prevention of cerebrovascular disease. However, its role in prevention of dementia in the elderly remains inconclusive. Some studies confirmed that statins can prevent dementia[13–15], but others disagreed [16–18].To the best of our knowledge, there has been no study to assess the efficacy of statins in preventing subsequent dementia in patients with LOD.

The growing burden of LOD and dementia in the aging society all over the world makes it mandatory to investigate the impact of statins on dementia in patients with LOD. We aimed to investigate whether the statins can reduce the subsequent onset of dementia in patients with LOD by performing a population-based retrospective cohort analysis.

## Methods

### Classification of evidence

This cohort study provides Class III evidence that statins (for at least 3 month) is effective in preventing dementia, conducted after an average of 6.1 years of follow-up in participants who were aged ≧65 years with a history of LOD (Hazard Ratio = 0.674, 95% CI 0.547–0.832).

### Data sources and study population

We used the Longitudinal Health Insurance Database (LHID) 2005 collated from the National Health Insurance Research Database (NHIRD) released by the National Health Research Institute in Taiwan. The National Health Insurance program finances the health care for 99% of the residents of Taiwan (>25 million enrollees). LHID 2005 contains all the original claims data of 1,000,000 beneficiaries enrolled in the year 2005. We selected the data, by random sampling, from the 2005 Registry for Beneficiaries (ID) of the NHIRD, in which registration data of every beneficiary of the National Health Insurance program during the period from January 1, 2005 to January 1, 2006 are recorded. The LHID2005 includes comprehensive information on the insured people, including demographic data, dates of clinical visits, diagnostic codes, details of prescriptions, expenditure levels and dates of enrolment and withdrawal between January 1996 and December 2009. There was no significant difference in the gender distribution (χ^2^ = 0.008, *df* = 1, *P*-value = 0.931) between the enrollees listed in the LHID2005 and those originally enrolled under NHIRD. (http://nhird.nhri.org.tw/en)

Codes from International Classification of Diseases, 9th revision, Clinical Modification (ICD-9-CM) are used in LHID2005. Personal information, including family history of cancer, lifestyle factors, and habits such as smoking and alcohol use, are not available from the NHIRD.

The dataset used in this study consists of de-identified secondary data released to the public for research purposes. This study was approved by the Institutional Review Board of Taipei City Hospital, Taiwan.

#### Study population

We conducted a retrospective cohort study from January 1, 1996 to December 31, 2009. We enrolled 45,973 individuals aged ≥65 years into our study population from the 1,000,000 beneficiaries in the LHID 2005.^6^

#### Identification of LOD, dementia and lipid lowering agents (LLAs)—taking history

The study subjects aged ≥65 years who had filed at least three service claims between 1996 and 2009 for either outpatient or inpatient care with a principal diagnosis of depression (ICD-9-CM: 311, 296.2, 296.3 and 300.4) were identified as patients with LOD. Similarly, those with a principal diagnosis of dementia (ICD-9-CM 331 290.0–290.4 and 294.1) were identified as patients with dementia. We excluded the patients who had depression before 65 years of age and those who were not treated with anti-depressants. “LLAs use” was defined as the use of lipid lowering agents(LLAs) and divided into three groups–statins users, fibrates users and users of other LLAs. The observation duration is during the entire study period in patients without dementia, or until dementia occurred. Patients with dementia occurred less than 3 months after the diagnosis of depression were excluded.

#### Demographic variables and comorbidities

The demographic variables under study included age, gender, and the presence or absence of diabetes mellitus (DM) (ICD9-CM: 250), hypertension (HTN) (ICD9-CM: 401–405), chronic obstructive pulmonary disease (COPD) (ICD9-CM: 490–496), chronic renal insufficiency (CRI) (ICD9-CM 585), ischemic heart disease (IHD) (ICD9-CM:410–414), congestive heart failure (CHF) (ICD9-CM:428–429), and cerebrovascular accident (CVA) (ICD9-CM:430–436).

### Statistical analysis

All patients enrolled were followed until the end of 2009 unless one of the following occurred: diagnosis of dementia being established, death, or dropout from the National Health Insurance program. We estimated the risk of dementia among the study cohort using age- and gender-adjusted SIRs. The 95% CIs for the SIRs were estimated under the assumption that the observed number of dementia cases followed a Poisson probability distribution. Categorical variables were compared using the χ^2^ test between groups of patients. The time dependent Cox proportional hazards model was applied for multivariate analyses to determine the adjusted hazard ratios (aHR) for dementia with statins use in patients with LOD. Propensity score methods was used to control the selection bias[19], and was derived from using binary logistic regression to figure out a propensity score for each patient who took statins or not. The demographic variables entered in the propensity model were as mentioned above. Subsequently, a one-to-one match between patients who received statins and those who did not receive statins was obtained using the nearest-neighbor matching method [19]. Kaplan-Meier estimate with Log rank test was used to measure the group of patients with dementia living beyond a specified period of time after diagnosis of LOD. All statistical analyses were performed with SAS (version 9.3; SAS Institute, Cary, NC). A *P*-value below 0.05 was considered statistically significant.

## Results

### Baseline characteristics and incidence of subsequent dementia between enrolled cases with or without LOD

In total, 45,973 patients aged ≧65 years were enrolled in this population study. **Table 1** shows the distributions of the demographic data and the comorbid medical disorders for the patients with LOD and their control cohort. Of the totally 45,973 patients, 5,952 (12.9%) had LOD. Patients with LOD were significantly younger, predominantly female, and had a higher prevalence of COPD, DM, HTN, IHD, CHF CVA, CRI and hyperlipidemia. Further, they were more likely to develop dementia during the follow-up period as compared with those without LOD (17.8% vs. 12.7%, *P* < 0.001). The age- and gender-adjusted SIRs of dementia for the enrolled cases with LOD relative to the control cohort was 2.785 (95% CI, 2.619–2.958). The mean follow-up duration was 13.33 ± 2.45 years for the subjects without LOD, and 6.03 ± 3.48 years for cases with LOD.

**Table 1:** The characteristics of study cohort with or without late-onset depression (n = 45,973).

		Without LOD		With LOD		P-value
		n = 40,021(%)		n = 5,952(%)		
Age (year)	Mean+/-SD	82.85+/-4.91		78.10+/-6.14		<0.001
Gender	F	18613	(46.5)	3413	(57.3)	<0.001
	M	21408	(53.5)	2539	(42.7)	
COPD	No	21032	(52.6)	2634	(44.3)	<0.001
	yes	18989	(47.4)	3318	(55.7)	
DM	No	28351	(70.8)	3681	(61.8)	<0.001
	yes	11670	(29.2)	2271	(38.2)	
HTN	No	10031	(25.1)	956	(16.1)	<0.001
	yes	29990	(74.9)	4996	(83.9)	
IHD	No	24198	(60.5)	2783	(46.8)	<0.001
	yes	15823	(39.5)	3169	(53.2)	
CHF	No	30430	(76.0)	4282	(71.9)	<0.001
	yes	9591	(24.0)	1670	(28.1)	
CVA	No	28243	(70.6)	3448	(57.9)	<0.001
	yes	11778	(29.4)	2504	(42.1)	
CRI	No	35782	(89.4)	5124	(86.1)	<0.001
	yes	4239	(10.6)	828	(13.9)	
Hyperlipidemia	No	24105	(60.2)	2602	(43.7)	<0.001
	Yes	15916	(39.8)	3350	(56.3)	
Dementia	No	34926	(87.3)	4892	(82.2)	<0.001
	yes	5095	(12.7)	1060	(17.8)	
Person-year	Mean+/-SD	13.33+/-2.45		6.03+/-3.48		<0.001

In patients aged ≧65 years, 12.9% were with late-onset depression.(LOD)

Age- and gender-adjusted standardized incidence ratios(SIR) of dementia for patients aged ≧65 years with LOD was 2.785 (95% CI 2.619–2.958).

Abbreviation: DM, diabetes mellitus; HTN, hypertension; COPD, chronic obstructive pulmonary disease; CRI, chronic renal insufficiency; IHD, ischemic heart disease; CHF, congestive heart failure; CVA, cerebrovascular accident; LOD, late-onset depression

### Baseline characteristics and incidence of subsequent dementia between patients of LOD with or without LLAs use

**Table 2** shows the distributions of the demographic data and comorbid medical disorders for the patients with LOD, compared between LLAs users and non-users. Of the 5,952 patients with LOD, 2,062 (34.6%) had LLAs medication. Patients using LLAs were significantly younger, predominantly female, and had a higher prevalence of DM, HTN, IHD, CHF, CVA, CRI and hyperlipidemia. There was lower prevalence of subsequent dementia in patients with LLAs use, compared with those without LLAs use before adjustment by χ^2^ test (15.2% vs. 19.2%, *P* <0.001). The mean follow-up duration for the subjects without using LLAs was 6.00 ± 3.50 years, and for cases with using LLAs was 6.10 ± 3.44 years.

**Table 2:** The characteristics of patients with late-onset depression, using or not using anti-lipid agents use (n = 5,952).

		Without anti-lipid agents		With anti-lipid agents		*P*-value
		n = 3,890(%)		n = 2,062(%)		
Age (year)	Mean+/-SD	78.45+/-6.37		77.43+/-5.63		0.779
Gender	F	2058	(52.9)	1355	(65.7)	<0.001
	M	1832	(47.1)	707	(34.3)	
COPD	No	1678	(43.1)	956	(46.4)	0.017
	yes	2212	(56.9)	1106	(53.6)	
DM	No	2763	(71.0)	918	(44.5)	<0.001
	yes	1127	(29.0)	1144	(55.5)	
HTN	No	820	(21.1)	136	(6.6)	<0.001
	yes	3070	(78.9)	1926	(93.4)	
IHD	No	2049	(52.7)	734	(35.6)	<0.001
	yes	1841	(47.3)	1328	(64.4)	
CHF	No	2883	(74.1)	1399	(67.8)	<0.001
	yes	1007	(25.9)	663	(32.2)	
CVA	No	2395	(61.6)	1053	(51.1)	<0.001
	yes	1495	(38.4)	1009	(48.9)	
CRI	No	3424	(88.0)	1700	(82.4)	<0.001
	yes	466	(12.0)	362	(17.6)	
hyperlipidemia	No	2602	(66.9)	0	(0.0)	<0.001
	yes	1288	(33.1)	2062	(100.0)	
Dementia	No	3144	(80.8)	1748	(84.8)	<0.001
	yes	746	(19.2)	314	(15.2)	
Person-year	Mean+/-SD	6.00+/-3.50		6.10+/-3.44		0.640

Abbreviation: DM, diabetes mellitus; HTN, hypertension; COPD, chronic obstructive pulmonary disease; CRI, chronic renal insufficiency; IHD, ischemic heart disease; CHF, congestive heart failure; CVA, cerebrovascular accident.

**Table 3** shows the distributions of the demographic data and comorbid medical disorders for the patients with LOD, classified according to whether they presented hyperlipidemia or not thereafter. Patients with LOD who presented hyperlipidemia had a higher prevalence of COPD, DM, HTN, IHD, CHF, CVA and CRI, but a lower prevalence of subsequent dementia.

**Table 3:** The characteristics of elderly patients with dementia in patients with late-onset depression, having or not having the diagnosis of hyperlipidemia (n = 5,952).

		Without hyperlipidemia		With hyperlipidemia		*P*-value
		n = 2602(%)		n = 3350(%)		
Age (year)	Mean+/-SD	78.57	(6.49)	77.73	(5.83)	<0.001
Gender	F	1345	51.7%	2068	61.7%	<0.001
	M	1257	48.3%	1282	38.3%	
COPD	No	1164	44.7%	1470	43.9%	0.510
	yes	1438	55.3%	1880	56.1%	
DM	No	1981	76.1%	1700	50.7%	<0.001
	yes	621	23.9%	1650	49.3%	
HTN	No	629	24.2%	327	9.8%	<0.001
	yes	1973	75.8%	3023	90.2%	
IHD	No	1484	57.0%	1299	38.8%	<0.001
	yes	1118	43.0%	2051	61.2%	
CHF	No	1964	75.5%	2318	69.2%	<0.001
	yes	638	24.5%	1032	30.8%	
CVA	No	1626	62.5%	1822	54.4%	<0.001
	yes	976	37.5%	1528	45.6%	
CRI	No	2364	90.9%	2760	82.4%	<0.001
	yes	238	9.1%	590	17.6%	
Dementia	No	2091	80.4%	2801	83.6%	<0.001
	yes	511	19.6%	549	16.4%	
Anti-lipid drugs	No	2602	100.0%	1288	38.4%	<0.001
	yes	0	.0%	2062	61.6%	

Abbreviation: DM, diabetes mellitus; HTN, hypertension; COPD, chronic obstructive pulmonary disease; CRI, chronic renal insufficiency; IHD, ischemic heart disease; CHF, congestive heart failure; CVA, cerebrovascular accident.

### Relative risk of dementia in LOD cohorts

Univariate time-dependent Cox proportional-hazards regression analysis was carried out to estimate the aHR of subsequent dementia in patients with LOD (**Table 4**). By controlling the potential confounders, including all the demographic variables, we found a significantly lower risk of dementia in patients using LLAs than in those who did not use LLAs (HR = 0.781, 95% CI 0.685–0.891, *p* < 0.001). The other independent risk factors were age, HTN, COPD, CRI, CVA and hyperlipidemia. However, Gender, DM, IHD, and CHF were not found to be the independent risk factors.

**Table 4:** Risk factors of dementia in patients with late-onset depression using univariate time dependent Cox proportional hazards model (n = 5,952).

	Hazard Ratios	95% CI	*P*-value
Anti-lipid drugs (Yes *v*.*s*. No)	0.781	0.685–0.891	<0.001
Age (Elder *v*.*s*.younger)	2.081	1.843–2.349	<0.001
Gender (Female *v*.*s*. male)	1.110	0.982–1.255	0.095
DM (Yes *v*.*s*. No)	1.120	0.991–1.266	0.069
HTN (Yes *v*.*s*. No)	1.439	1.190–1.738	<0.001
COPD (Yes *v*.*s*. No)	1.352	1.193–1.533	<0.001
CRI (Yes *v*.*s*. No)	1.177	1.001–1.385	0.049
IHD (Yes *v*.*s*. No)	1.126	0.997–1.271	0.056
CHF (Yes *v*.*s*. No)	1.118	0.982–1.273	0.093
CVA (Yes *v*.*s*. No)	1.708	1.513–1.928	<0.001
Lipid (Yes *v*.*s*. no)	0.821	0.728–0.926	0.001

Abbreviation: DM, diabetes mellitus; HTN, hypertension; COPD, chronic obstructive pulmonary disease; CRI, chronic renal insufficiency; IHD, ischemic heart disease; CHF, congestive heart failure; CVA, cerebrovascular accident.

Multivariate time-dependent Cox proportional-hazards regression analysis was carried out to estimate the aHR of subsequent dementia in patients with LOD (**Table 5**). By controlling the potential confounders, including all demographic variables, we also found a significantly lower risk of dementia in patients using statins than in those who did not use LLAs (HR = 0.674, 95% CI 0.547–0.832, *p* < 0.001). On the other hand, the patients taking a non-statin LLA (fibrate or other LLA) showed no significant reduction in the hazards risk of dementia as compared with those did not use LLAs (HR 0.826, P = 0.117, 95% CI 0.65–1.049; HR 0.724, P = 0.349, 95% CI 0.369–1.422).

The other independent risk factors were age, gender, DM, HTN, COPD and CVA. However, CRI, IHD, CHF and hyperlipidemia were not found to be independent risk factors.

**Table 5:** Risk factors of dementia in patients with late-onset depression using multivariate time dependent Cox proportional hazards model (n = 5,952).

	Hazard Ratios	95% CI	*P*-value
Anti-lipid drugs			0.003
Statins v.s. no	0.674	0.547–0.832	<0.001
Fibrate v.s. no	0.826	0.650–1.049	0.117
Others v.s. no	0.724	0.369–1.422	0.349
Age (Elder *v*.*s*. younger)	1.901	1.679–2.151	<0.001
Gender (Female *v*.*s*. male)	1.265	1.115–1.435	<0.001
DM (Yes *v*.*s*. No)	1.146	1.008–1.303	0.037
HTN (Yes *v*.*s*. No)	1.225	1.004–1.495	0.046
COPD (Yes *v*.*s*. No)	1.266	1.112–1.441	<0.001
CRI (Yes *v*.*s*. No)	1.113	0.942–1.314	0.207
IHD (Yes *v*.*s*. No)	1.034	0.907–1.178	0.617
CHF (Yes *v*.*s*. No)	0.949	0.827–1.088	0.450
CVA (Yes *v*.*s*. No)	1.597	1.410–1.809	<0.001
Lipid (Yes *v*.*s*. no)	0.980	0.808–1.188	0.835

Abbreviation: DM, diabetes mellitus; HTN, hypertension; COPD, chronic obstructive pulmonary disease; CRI, chronic renal insufficiency; IHD, ischemic heart disease; CHF, congestive heart failure; CVA, cerebrovascular accident.

### Factors associated with overall dementia-free survival after propensity score correction using the one-to-one nearest-neighbor matching method

**Fig 1:**
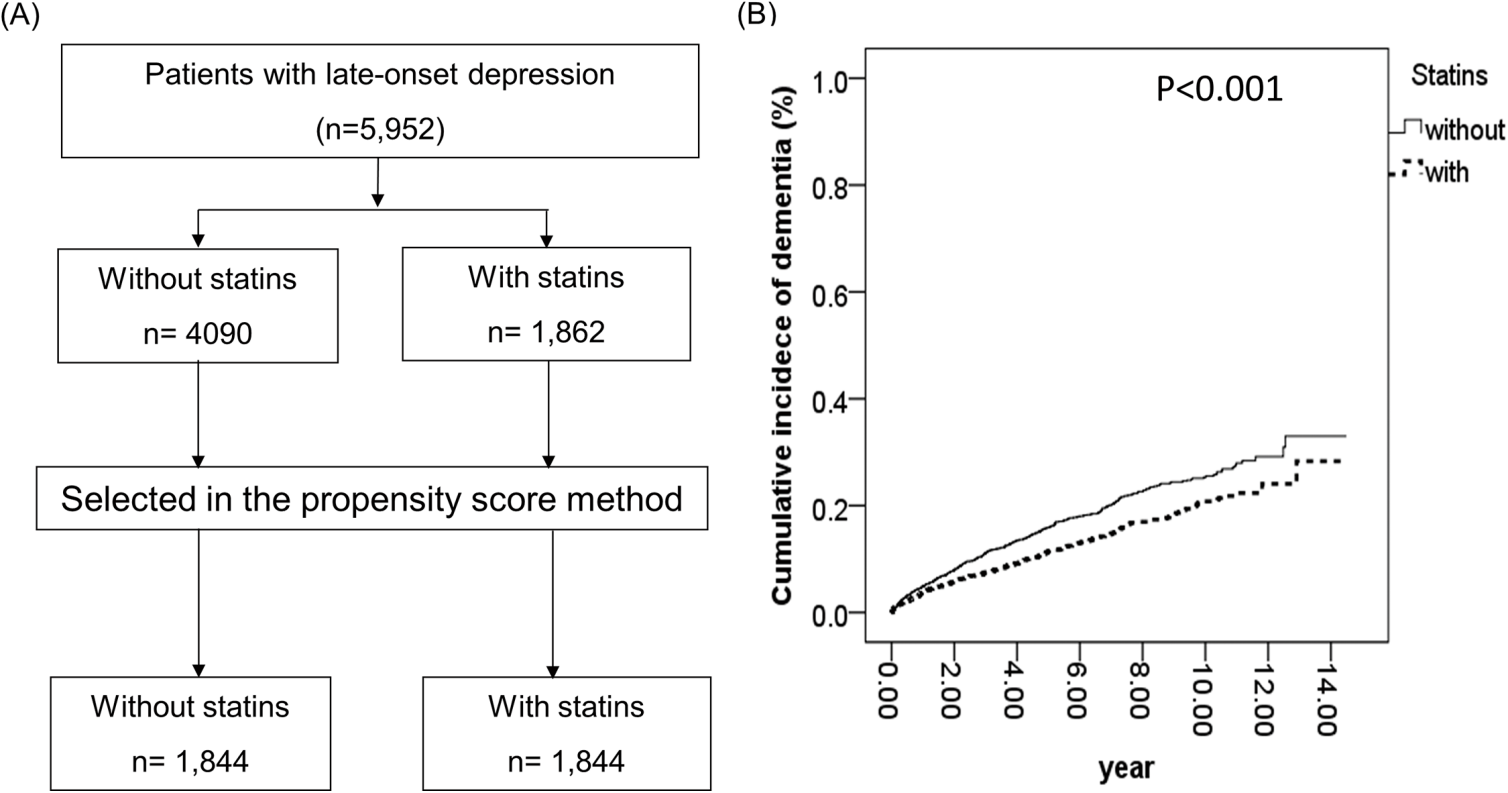
The effect of statins in reducing the risk of subsequent dementia in patients with late-onset depression. (A) Flowchart showing the matching process for statins users and non-users in the population studied. Finally, 1,844 pairs of matched patients were selected for analysis using the propensity score method, which minimizes interference by confounding factors. (B) Statins reduced the occurrence of subsequent dementia in patients with LOD (*p* < 0.001).

To validate the potential benefits of statins to patients with LOD in preventing dementia, we applied the propensity score correction method using one-to-one nearest-neighbor matching to minimize the interference of confounding factors, including all demographic variables (**Fig 1-A**). Since the patients who took statins might also have other known risk factors of dementia such as DM, hence, we needed to establish whether statins per se could prevent subsequent dementia. A total of 1,844 statins-users were selected to match the counterpart number of non-users of statins for comparison of the influence of the aforementioned factors, which appeared well-matched between groups (**Table 6**). The subsequent multivariate analysis revealed that statins could significantly decrease the risk of dementia (**Fig 1-B**) (*P* <0.001), and showed to be an independent protective factor against dementia (**Table 7**). The aHR for statins use was 0.717 (95% CI 0.612–0.840, *P* <0.001). Only age, gender, DM, COPD and CVA remained as the independent risk factors of dementia after adjustment of variables in this analysis.

**Table 6:** The Comparison of the baseline demographics of patients with late-onset depression after matching by propensity score method (n = 3,688).

		Without statins		With statins		*P*-value
		N = 1,844(%)		N = 1,844(%)		
Age (year)	<75	1294	70.2%	1296	70.3%	0.943
	> = 75	550	29.8%	548	29.7%	
Sex	F	1215	65.9%	1222	66.3%	0.808
	M	629	34.1%	622	33.7%	
COPD	No	831	45.1%	845	45.8%	0.643
	yes	1013	54.9%	999	54.2%	
DM	No	939	50.9%	920	49.9%	0.531
	yes	905	49.1%	924	50.1%	
HTN	No	147	8.0%	156	8.5%	0.589
	yes	1697	92.0%	1688	91.5%	
IHD	No	653	35.4%	674	36.6%	0.471
	yes	1191	64.6%	1170	63.4%	
CHF	No	1265	68.6%	1275	69.1%	0.722
	yes	579	31.4%	569	30.9%	
CVA	No	977	53.0%	978	53.0%	0.974
	yes	867	47.0%	866	47.0%	
CRI	No	1563	84.8%	1547	83.9%	0.469
	yes	281	15.2%	297	16.1%	

Abbreviation: DM, diabetes mellitus; HTN, hypertension; COPD, chronic obstructive pulmonary disease; CRI, chronic renal insufficiency; IHD, ischemic heart disease; CHF, congestive heart failure; CVA, cerebrovascular accident.

**Table 7:** The effect of statin in preventing dementia in patients with late-onset depression, evaluated by using multivariate Cox proportional hazards model after matching by propensity score (n = 3,688).

	Hazard Ratios	95% CI	*P*-value
Statin (Yes *v*.*s*. No)	0.717	0.612–0.840	<0.001
Age (Elder *v*.*s*. younger)	1.825	1.551–2.148	<0.001
Sex (Female *v*.*s*. male)	1.274	1.074–1.512	0.005
DM (Yes *v*.*s*. No)	1.227	1.047–1.437	0.011
HTN (Yes *v*.*s*. No)	1.414	0.960–2.084	0.080
COPD (Yes *v*.*s*. No)	1.405	1.188–1.662	<0.001
CRI (Yes *v*.*s*. No)	1.061	0.863–1.303	0.575
IHD (Yes *v*.*s*. No)	1.177	0.983–1.408	0.076
CHF (Yes *v*.*s*. No)	0.895	0.753–1.064	0.208
CVA (Yes *v*.*s*. No)	1.616	1.375–1.899	<0.001

Abbreviation: DM, diabetes mellitus; HTN, hypertension; COPD, chronic obstructive pulmonary disease; CRI, chronic renal insufficiency; IHD, ischemic heart disease; CHF, congestive heart failure; CVA, cerebrovascular accident.

## Discussion

To the best of our knowledge, this is the first study to demonstrate that statins can reduce the subsequent risk of dementia in patients with LOD, after adjusting for multiple confounding comorbidities.

To our knowledge, there is no study investigating the role of statins in preventing cognitive decline or subsequent dementia in patients with LOD. However, conclusions from previous prospective investigations on the effects of statins in the cognitive functioning of the aged (not limited to those with LOD) are controversial; the reported effects can be positive[13–15, 20], neutral[16–18], or negative [21, 22], far from reaching any consensus. In nested case-control studies conducted by Jick et al., statins users showed to run a significantly lower risk of dementia (OR 0.29, CI 0.13 to 0.63) than non-users[13]. Zamrini et al. also demonstrated that statins users benefited a 39% lower risk of Alzheimer’s dementia when compared with non-users (OR 0.61, 95% CI 0.42 to 0.87)[14]. The Wolozin study was a cross-sectional analysis and found a significantly reduced prevalence of Alzheimer’s dementia in 69.3% of all statins users as a whole [20]. Hajjae et al. found a lower risk of Alzheimer’s dementia and vascular dementia in people who took statins (P = 0.01) in their clinic based study [15]. However, some other studies questioned the hypothesis that statins has a protective effect to dementia [16–18], or even inversely reported its potential detrimental effects on cognitive performance [21, 22].

The most important finding from our study was that only statins users could benefit from the reduced risk of the subsequent onset of dementia in patients with LOD, while other non-statin LLAs users did not. In our current study, there was a 33% reduction in the risk of dementia in patients with LOD associated with ongoing statins use as compared with the non-users of LLAs; the difference was significant (P<0.001, 95% CI 0.54 7–0.832). When comparing the patients taking a non-statin LLA (fibrate or other LLA) with those who did not use LLAs, we found no significant difference or reduction in their hazards risk of dementia. The finding was similar to Jick’s report[13] that the patients ≥50 y/o who were prescribed statins had a substantially lowered risk of developing dementia, but patients prescribed non-statin LLAs did not have a reduced risk for dementia. In contrast, the Rockwood’s CSHA study demonstrated that the use of statins and other LLAs could both reduce risk of AD in subjects younger than 80 years of age[23]. Nevertheless, the populations of both studies were not limited to LOD patients.

In addition, our study also agrees with the previous findings that LOD is a risk factor of the subsequent dementia. In our current study, the prevalence of LOD among the elderly in Taiwan was 12.9%, which was close to the value (13.5%) reported by a previous meta-analysis[3]. The age- and gender-adjusted SIR of dementia in patients with LOD, compared with their control cohort, was 2.785 (95% CI 2.619–2.958), which was also similar to Buntinx’s report[24]. In their retrospective cohort study, the HR for patients’ ≥50 y/o with LOD to have dementia was 2.55 when compared with those without LOD (95% CI 1.19–5.47).

Patients with LOD are reported to run a higher risk of having vascular morbidities with concomitant cognitive impairment [11, 25–28]. There are mounting evidences to suggest a relation between lipids and vascular factors involving the brain in dementia. Hofman et al. found a link between vascular risk factors and dementia in their epidemiological studies[29]; Snowdon el al. reported that very small strokes can precipitate clinical dementia in cognitively normal elderly people with Alzheimer’s disease pathology[30]; Frears et al. found the effect in cell culture of cholesterol on degradation of the amyloid-β peptide [31]; Buee el al. reported the abnormal appearance of microvascular endothelial cells in affected brain areas in Alzheimer’s disease[32]; and Li el at. found a possible role of the LDL receptor-related protein in Alzheimer’s disease[33]. However, the precise mechanisms by which any or all of these lipid and vascular factors might be associated with dementia in patients with LOD are at present poorly understood.

Several mechanisms may be related to the potential effects of statins in preventing subsequent dementia in patients with LOD. Statins (3-hydroxy-3-methylglutaryl coenzyme A reductase inhibitors) may modify lipid and vascular risk factors through their action on lowering cholesterol and also through other non-cholesterol lowering effects. Statins, acting via a cholesterol-dependent mechanism, might reduce the production of amyloid-β peptide[34] and NFT burden[33] to have neuroprotective effects in patients with Alzheimer’s disease (AD).

The non-cholesterol lowering actions of statins include their actions on nitric oxide, platelet adhesion and anti-inflammatory action. Firstly, they can improve the endothelial function of atherosclerotic vessels and increase nitric oxide[35] to relax the vascular smooth muscles leading to vasodilatation, thus to play a role in reducing AD pathology[36, 37]. Secondly, statins can prevent vascular plaque formation via their actions to inhibit platelet adhesion as they decrease plasminogen activator levels and suppress inflammation effects [38], which can partially explain why our study had a positive finding. Because the patients with LOD have more vascular risk factors to increase the likelihood of developing dementia, the use of statins may give them a beneficial effect in preventing dementia by reducing the risk of cerebrovascular disease via modifying lipid and vascular risk factors. Furthermore, statin may prevent depression by lower LDL and inflammation status. There are evidences showing the associations between LDL and inflammation and further depression.[39, 40]

Taken together, statins use might be a potentially beneficial strategy for the prevention of subsequent dementia (or cognitive impairment) in patients with LOD. Since this is an observational study, our results need to be duplicated using other populations, and double-blind randomized controlled trials are warranted to further investigate how the clinical use of statins may prevent dementia after LOD.

Although our study has the strengths such as a large number of patients enrolled, long follow-up period and detailed records of co-morbid diseases, it has the following limitations: 1) Several factors affecting LOD, such as lifestyle, obesity, smoking, alcohol use, the use of vitamin or fish oil supplementation, and family history, could not be assessed from this dataset. In addition, some other residual confounders that may exert their effects were not taken into account; 2) The subtypes of dementia were not revealed by the dataset, so we could not determine whether the protective effects of statins were kept the same in different subtypes of dementia; 3) Information on some important depression-related variables, such as the severity of depression, the frequency of depressive episodes and the comorbid psychotic features, was not available.

## Conclusions

The evidence presented in our study provides a window into another potential indication for statins use as a treatment option in patients with LOD. Selective use of statins in patients with LOD could be implemented to reduce the risk of subsequent dementia.
